# Anesthetic Considerations for Patients With Mitral Stenosis Undergoing Orthotopic Liver Transplant

**DOI:** 10.7759/cureus.47751

**Published:** 2023-10-26

**Authors:** Justin Mitchell, Amar Alnemer, Selina Deiparine, Erica Stein, Leonid Gorelik

**Affiliations:** 1 Anesthesiology, University of California, Los Angeles (UCLA) Medical Center, Los Angeles, USA; 2 Anesthesiology, The Ohio State University College of Medicine, Columbus, USA; 3 Orthopedic Surgery, University of Miami Hospital, Miami, USA; 4 Anesthesiology, The Ohio State University Wexner Medical Center, Columbus, USA

**Keywords:** mitral valve stenosis, anesthesiology, liver transplant anesthesia, cardiothoracic anesthesia, liver transplant

## Abstract

A 70-year-old male presented for an orthotopic liver transplant (OLT) with co-existing moderate-severe mitral valve stenosis. The hemodynamic goals of managing mitral stenosis posed a significant additional challenge to this patient’s care. Intraoperative transesophageal echocardiography (TEE) was critical in guiding volume status and resuscitation. In addition, the patient's valvulopathy guided our vasoactive medication selection and arrhythmia prevention. In this article, we describe the multidisciplinary discussions regarding preoperative valvular intervention as well as the intraoperative techniques used to preserve cardiac output while avoiding coagulopathy and arrhythmias. We discuss the pathophysiology of valvular disease in the context of liver failure and the guidelines by which this disease process is classified. In addition, we discuss the benefits and limitations of intraoperative TEE in evaluating this unique physiology.

## Introduction

Mitral stenosis increases morbidity and mortality during the peri- and post-operative periods [1]. Mitral stenosis results in increased resistance to left ventricular filling and subsequent deleterious effects including pulmonary congestion, increased pulmonary vascular resistance, increased right ventricular wall tension, and hepatic congestion [2,3]. The presence and severity of mitral stenosis should be noted in patients with end-stage liver disease. When these patients present for liver transplantation, their hemodynamic management can pose a challenge to anesthesiologists due to large volume shifts, congestive back pressure secondary to right heart failure physiology that causes an increase in inferior vena cava (IVC) venous pressure that is transmitted to the hepatic venous system, and the transfusion burden secondary to significant blood loss. Their management is further complicated during liver reperfusion when bradycardia and other arrhythmias that are especially deleterious for mitral stenosis physiology can occur. We present a case of a patient with hemodynamically significant mitral stenosis undergoing an orthotopic liver transplant (OLT) and discuss the challenges for anesthesiologists. To our knowledge, this is the first case report of this valvular pathophysiology during a liver transplant.

## Case presentation

A 70-year-old male with a history of nonalcoholic steatohepatitis cirrhosis requiring weekly paracentesis, model for end-stage liver disease 27, aortic stenosis status post transcatheter aortic valve replacement, coronary artery disease, and hypertension presented for OLT. On initial evaluation, he was alert and oriented without acute distress. He endorsed minimal fatigue that had been present for several months. He was diagnosed with cirrhosis by computed tomography a year previously, with his first decompensation episode of ascites two months later.

Three months prior to transplantation, a transthoracic echocardiogram (TTE) revealed a left ventricular ejection fraction (LVEF) of 55-60% and irregular septal motion consistent with a left bundle branch block. The bioprosthetic aortic valve was well seated with a trace paravalvular leak. However, the echocardiogram also revealed severe posterior annular calcification of the mitral valve and moderate-to-severe mitral stenosis with a mean gradient of 10 mmHg at a heart rate of 80 beats per minute (bpm). Preoperative pulmonary artery pressure was 26/10 mmHg, with a mean pressure of 17 mmHg. Measured by transesophageal echocardiography (TEE), cardiac output was 10.9 L/min and cardiac index was 4.8 L/min/m2. A multidisciplinary team of anesthesiologists, cardiologists, and cardiothoracic surgeons concluded that this patient was not a candidate for open heart surgery nor would a mitral valve balloon valvuloplasty be beneficial given evidence of mitral valve opening on TTE. The consensus decision was made to proceed with OLT.

**Figure 1:**
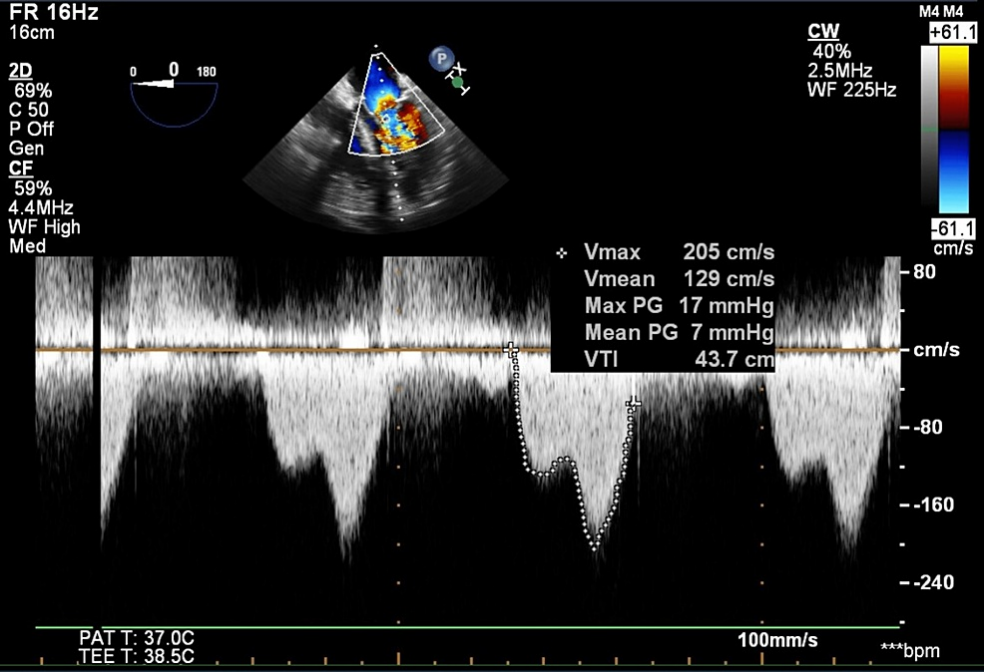
Intraoperative TEE image demonstrating mitral stenosis

On preoperative exam, the patient had mild bilateral peripheral edema to the level of the ankles and mild rhonchi. A diastolic murmur was also appreciated. His vitals were as follows: (1) non-invasive blood pressure of 121/62, (2) heart rate of 72, and (3) respiratory rate of 16. We placed a pre-induction arterial line due to his valvular pathophysiology. Since arrhythmias that can occur during liver transplantation, such as atrial fibrillation, can be catastrophic to a patient with mitral stenosis, we placed defibrillator pads on the patient prior to induction. We kept them connected to the defibrillator throughout the case and had a bolus dose of amiodarone readily available in the event the patient developed an unstable arrhythmia. General anesthesia was induced via propofol and fentanyl, in order to avoid significant tachycardia due to intubation response. We prophylactically administered phenylephrine during induction in order to maintain systemic vascular resistance and avoid hypotension. Intraoperative TEE revealed an LVEF of 50-55% without any regional wall motion abnormalities. The structure and function of the bioprosthetic aortic valve were unchanged, and the pulmonic and tricuspid valves were unremarkable. The mitral valve leaflets were restricted and calcified with a mean gradient of 7 mmHg at a heart rate of 86 bpm (Figure 1). The area of the mitral valve was calculated to be 1.89 cm2 by the continuity equation. Due to the presence of significant epigastric varices, a transgastric basal short axis view of the mitral valve was not obtained. In addition, the left atrium appeared significantly enlarged, measuring 7.4 cm. We avoided significant tachycardia by using adequate pain control and closely monitoring the patient’s volume status and estimated blood loss. We selected vasopressors and inotropes with limited beta agonist effect (vasopressin, phenylephrine, and norepinephrine) to avoid worsening the patient’s mitral stenosis physiology. Intraoperative pulmonary arterial catheterization revealed pulmonary artery pressures of 40-60/20-31 mmHg (mean pulmonary artery pressures of 30-35), central venous pressures of 15-25 mmHg, and mean arterial pressures in the range of 50-60 mmHg. The intraoperative cardiac output was 11 L/min and the cardiac index was 4.8 L/min/m2, measured using the thermodilution method. The patient did not have any previous abdominal surgery, which helped reduce blood loss during dissection. In addition, point-of-care viscoelastic testing was performed with rotational thromboelastometry to guide transfusion. The patient received eight units of packed red blood cells, three units of fresh frozen plasma, two units of cryoprecipitate, and one pool of platelets throughout the surgery. We also had a low threshold to administer concentrated factors such as prothrombin complex concentrate and fibrinogen concentrate if the patient began to show signs of volume overload but remained coagulopathic.

The OLT proceeded without complication and the patient tolerated hepatic reperfusion without any significant hemodynamic perturbations. Following the OLT, the patient remained hemodynamically stable in the ICU. He was extubated on postoperative day 1. His postoperative course was complicated by persistent hyperbilirubinemia. An endoscopic retrograde cholangiopancreatography was performed, showing an anastomotic stricture, and a stent was placed. The hyperbilirubinemia resolved and his LFTs normalized. He was discharged home on postoperative day 14. An echocardiogram performed approximately two months after the transplant revealed an LVEF of 60-65% with anteroseptal hypokinesis and mild atrial enlargement. There was continued evidence of moderate mitral stenosis with a mean gradient of 6 mmHg. Two years following the transplant, the patient was doing well with immunosuppression and regular follow-up. He has not required a mitral valve replacement.

## Discussion

The diagnosis of mitral stenosis with elevated transmitral gradients can be deceiving given the hypervolemia and hyperdynamic circulation associated with liver failure. Preoperatively, our patient’s gradient was measured echocardiographically as 10 mmHg, qualifying him for moderate to severe mitral stenosis per the American Society of Echocardiography Guidelines [4]. However, the intraoperative area of the mitral valve was calculated to be 1.89 cm2 by the continuity equation, qualifying him for mild mitral stenosis. Unfortunately, due to the presence of significant epigastric varices, a transgastric basal short axis view of the mitral valve was unable to be evaluated to directly measure the mitral valve area. This point in our case emphasizes the concept of pseudo-severe mitral stenosis, where there is mild structural mitral stenosis, but the physiology of liver failure, including hypervolemia and hyperdynamic circulation, increases the transmitral gradient into the severe range [5].

OLT presents multiple challenges for anesthesiologists. The numerous components of this complex surgery include major vascular manipulations, coagulopathy, rapidly changing preload and afterload conditions, electrolyte and acid-base imbalances, significant blood component transfusions, hypothermia, and pharmacologic hemodynamic support [6,7]. For OLT to be successful, optimal cardiac performance is essential. Patients with valvular dysfunction, especially mitral stenosis, require heightened attention related to (1) major blood loss, (2) decreased preload after clamping, and (3) increased preload after unclamping of the inferior caval and portal veins, predisposing to potential hyperdynamic overload [8]. In order to minimize these complications as well as maintain hemodynamic stability for this patient, our team had extensive discussions with the surgical team, stressing the importance of minimizing blood loss if possible and avoiding large hemodynamic swings. The surgical technique included the application of a partial IVC clamp and piggyback anastomosis. The use of this technique allows the maintenance of partial blood flow through the IVC during the anhepatic phase, which can improve the hemodynamic stability during this phase as well as during reperfusion. While veno-venous bypass can be useful in certain hemodynamically unstable situations, it was not utilized in this case. In addition, the risk of transfusion-related hypocalcemia, citrate load, hypothermia, hyperkalemia, inflammatory assaults, arrhythmias, and postreperfusion syndrome must be considered [9]. The pathophysiology of mitral stenosis remains relevant once the liver allograft is implanted. As the mitral valve had become stenosed to a mitral valve area of less <2 cm2 (from a normal mitral valve area of 4-6 cm2) and the diastolic pressure gradient between the left atrium and the left ventricle developed, there are now chronically elevated left atrial pressures hindering forward flow. This backlogged flow was then transmitted to the pulmonary vasculature, resulting in a cycle of chronic vasoconstriction, sustained pulmonary hypertension, and right atrial and right ventricular remodeling. This increased congestion and pressure places the newly transplanted liver allograft at risk for failure secondary to oxidative injury, decreased perfusion, and impaired waste excretion [10,11].

The surgical options for patients with mitral stenosis in need of OLT are as follows: (1) repair the mitral valve prior to OLT, (2) undergo mitral valve replacement and OLT simultaneously, or (3) undergo OLT prior to mitral valve replacement [12-14]. Centers often require option 1, because of the hemodynamic sequelae of mitral stenosis discussed above [12]. There is not a significant amount of data on option 2. According to one analysis, a specific subset of patients can safely undergo simultaneous operations, specifically those who are Child-Pugh Class B and have normal ventricular function and no cardiac surgical history [14]. Our patient pursued option 3. According to the 2020 American College of Cardiology/American Heart Association Guidelines for the management of patients with valvular heart disease, surgical intervention for non-rheumatic mitral stenosis is not pursued until symptoms become severely debilitating and are refractory to heart rate control and diuresis [13].

Evaluation of mitral stenosis should be done using joint American Society of Echocardiography and European Association of Echocardiography Guidelines [4]. The parameters of importance when screening for mitral stenosis include the mean diastolic transmitral pressure gradient, mitral valve area measurements, and reactive changes including left atrial dilation, right ventricular hypertrophy, and elevated right ventricular pressures. On average, a mean gradient of >10 mmHg is consistent with severe mitral stenosis, 5-10 mmHg with moderate mitral stenosis, and <5 mmHg with mild mitral stenosis [15,16]. A nationwide task force sought to evaluate the risks and benefits of TEE and determined that the benefit of acute diagnosis and management of potentially life-threatening conditions such as myocardial stunning, intracardiac thrombosis pulmonary embolus, and left ventricular outflow tract obstruction outweigh the risk of gastrointestinal bleeding or potential variceal bleeding [17]. This task force noted strong impressions that hypotension caused by myocardial dysfunction, especially from right ventricular failure, was often missed when using conventional monitors and was more reliably identified by TEE imaging. The three-dimensional imaging groups were also shown to have better overall outcomes compared to the two-dimensional [18-19]. Furthermore, using TEE-derived intravascular volume to manage blood pressure during full or partial IVC cross-clamping was considered more reliable than other invasive monitors [18]. During the anhepatic stage, TEE images were reported to provide better information in distinguishing between two common causes of hypotension: inadequate venous return and left ventricular outflow tract obstruction. Overall, the additional information enabled by three-dimensional imaging is thought to result in better management of mitral stenosis [19,20]. While TEE can be useful and informative in any liver transplant, it is especially helpful in the setting of hemodynamic management of mitral stenosis patients undergoing this complex transplant.

## Conclusions

In conclusion, this case of a 70-year-old male undergoing an orthotropic liver transplant illustrates several important considerations for the management of mitral stenosis in patients undergoing such procedures. Intraoperative interventions aimed at preventing backflow to the graft should be considered, with the administration of blood products versus factor concentrates as appropriate to avoid volume overload. Other interventions, such as the choice of pressor, avoidance and treatment of deleterious arrhythmias, and maintenance of preload and afterload during the surgery's most hemodynamically unstable times are crucial to the intraoperative care of these patients. Most importantly, proper planning and communication with the surgical team prior to surgery is critical for maintaining cardiovascular stability. We recommend detailed characterization of cardiovascular and valvular function by preoperative transthoracic echocardiography using gradients as well as planimetry and intraoperative TEE for dynamic monitoring during the procedure. This is especially crucial in patients with suspected valvulopathy. It is important to recognize that the hyperdynamic state in cirrhosis complicates the assessment of valvular function. Following the transplant, when the hyperdynamic state resolves, the severity of valvular disease may improve. The combination of preoperative planning, surgical technique, and hemodynamic management guided by TEE can lead to a successful transplant and good graft function in patients with complex valvular pathophysiology.
